# Human Motion Enhancement and Restoration via Unconstrained Human Structure Learning

**DOI:** 10.3390/s24103123

**Published:** 2024-05-14

**Authors:** Tianjia He, Tianyuan Yang, Shin’ichi Konomi

**Affiliations:** 1 Graduate School of Information Science and Electrical Engineering, Kyushu University, Fukuoka 819-0395, Japan; 2 Faculty of Arts and Science, Kyushu University, Fukuoka 819-0395, Japan

**Keywords:** motion capture, human motion data, motion enhancement, denoising, graph convolutional networks, self-attention

## Abstract

Human motion capture technology, which leverages sensors to track the movement trajectories of key skeleton points, has been progressively transitioning from industrial applications to broader civilian applications in recent years. It finds extensive use in fields such as game development, digital human modeling, and sport science. However, the affordability of these sensors often compromises the accuracy of motion data. Low-cost motion capture methods often lead to errors in the captured motion data. We introduce a novel approach for human motion reconstruction and enhancement using spatio-temporal attention-based graph convolutional networks (ST-ATGCNs), which efficiently learn the human skeleton structure and the motion logic without requiring prior human kinematic knowledge. This method enables unsupervised motion data restoration and significantly reduces the costs associated with obtaining precise motion capture data. Our experiments, conducted on two extensive motion datasets and with real motion capture sensors such as the SONY (Tokyo, Japan) mocopi, demonstrate the method’s effectiveness in enhancing the quality of low-precision motion capture data. The experiments indicate the ST-ATGCN’s potential to improve both the accessibility and accuracy of motion capture technology.

## 1. Introduction

Motion capture technology employs either wearable or optical sensors to capture genuine human movements. The basic principle of motion capture sensors centers on recording the trajectory of human movement within a real space, capturing data at specific intervals. These motion data can then be mapped onto a virtual human model in the computer’s digital space, thereby reproducing authentic human actions within virtual environments. Compared to manually constructed human motions, real human movement data collected using motion capture sensors can more accurately replicate the physical properties and biomechanical principles of human motion. Consequently, the visualized movements are rendered more naturally and fluidly. Due to its complexity and cost, motion capture technology has historically been utilized primarily for industrial-grade movie special effects and the creation of virtual characters in large video games. This technology is generally inaccessible to the ordinary user.

Industrial-grade motion capture sensors, characterized by their complexity and high cost, demand stringent conditions for both the operational environment and the individuals performing the motions [1]. Despite these requirements, they provide data of exceptional precision. As motion capture applications have broadened, consumer-level motion capture sensors have emerged, becoming increasingly prevalent [2,3]. These sensors have found applications across various fields, including independent game developments on a smaller scale, the creation of virtual digital human models, and the gathering and analysis of medical data of the human skeleton [4,5,6]. Notably, these consumer-grade sensors are designed to be less complex, offering more flexibility in terms of operational scenarios and procedures without the need for elaborate setups. Nonetheless, the affordability of these sensors comes at the cost of reduced precision in motion data capture. This reduction in accuracy may result in various issues, such as distortions, missing details, or even noticeable errors within the replicated human motions.

The accuracy of sensors is influenced by several factors, including the following: 1. Random noise inherent in the data collection process, which is an unavoidable aspect of sensor technology. 2. External environmental conditions that can introduce interferences, such as variable lighting conditions and electromagnetic disturbances, potentially compromising the integrity of the data collected. 3. Algorithmic errors, particularly from the integration of data from accelerometers and gyroscopes; these errors tend to accumulate over time, progressively degrading the quality of the motion data [7]. 4. Displacement or movement of the sensors themselves during data collection, which can occur if the sensors are not securely attached to the skeleton [8]. 5. Data omissions or inaccuracies resulting from optical sensors being obstructed or misaligned, leading to the loss or incorrect estimation of key points; Due to the limited precision and number of sensors in low-cost motion capture systems, these errors are more pronounced and frequent.

To enhance the quality of such sensor data, related research generally falls into two categories: (1) reducing data errors through methods such as filtering during the data collection process to achieve smoother results [9]; and (2) denoising and enhancing fully collected data, such as human motion sequences derived from motion capture sensor data [10]. This study focused on the latter approach, as methods for processing fully collected motion data can be applied across a wider range of sensor types, offering better universality. In contrast, the former approach requires the adoption of different filtering strategies tailored to various types of sensors.

Using graph convolutional networks (GCNs) to process human motion data is a very natural idea, considering that the human skeletal structure can intuitively be viewed as a graph structure [11]. Studies employing GCN-based methods for human pose classification and human motion synthesis have achieved commendable results [12,13,14,15]. However, applications in human motion enhancement are relatively scarce. Although traditional GCNs have demonstrated effective outcomes in the domain of human motion, there still exist two limitations. First, GCN-based methods typically integrate with Recurrent Neural Networks (RNNs) or Temporal Convolutional Networks (TCNs) to handle motion sequences. However, the recurrent structure of RNNs leads to forgetting distant sequence information as the sequence length increases, performing poorly in long-sequence experiments [16]. Moreover, the inability to parallel process due to the recurrent structure results in low computational efficiency. While TCNs offer higher computational efficiency, they are limited by the need for manually setting the size of the receptive field, which cannot flexibly handle motion sequences of varying lengths [17]. To address this issue, we designed a temporal self-attention (TSA) module that computes attention across different time steps to capture dependencies between preceding and subsequent motions. Compared to TCNs, TSA offers a more flexible global receptive field and, due to its parallel computation mode, higher computational efficiency compared to those of RNNs.

**Figure 1:**
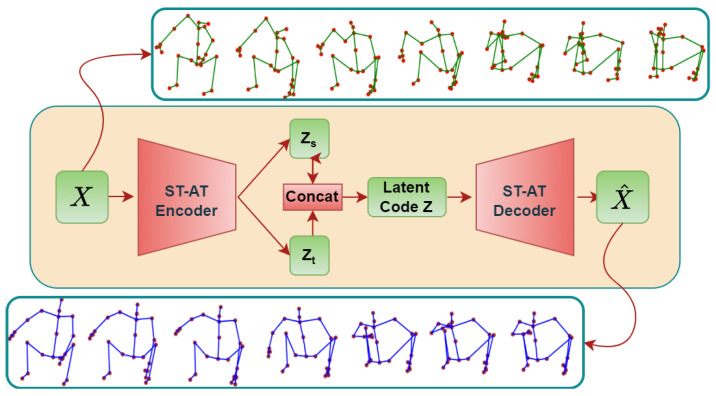
Design of overall framework structure for ST-ATGCN.

The second limitation is the significant decline in the information transmission efficiency of traditional GCNs as the number of nodes in the human skeletal graph increases, with nodes further apart having a weaker ability to communicate. This mechanism limits the ability of GCNs to learn the human skeletal graph, because unlike traditional graph data, nodes that are far apart in the human skeletal graph are strongly connected in many instances. To address the issue of inefficient propagation efficiency that hinders the understanding of human motions by GCNs, some studies have attempted to impose additional constraints on the model based on prior knowledge of human kinematics. To overcome this problem, our spatio-temporal attention-based GCN (ST-ATGCN), as illustrated in Figure 1, utilizes a learnable parameter matrix based on the self-attention mechanism, to replace the inherent adjacency matrix. This module, SA-GC, by integrating a learnable shared parameter matrix with attention maps of nodes across all different motions, learns human motion patterns, thereby overcoming the limitations brought by the traditional GCN’s information transmission mechanism. This design enables the ST-ATGCN to effectively understand the human skeletal structure without the need for additional human kinematic prior knowledge.

Many spatio-temporal models adopt a dual-stream architecture, wherein distinct networks are utilized to process spatial and temporal data. However, in reconstruction tasks, this dual-stream design confronts challenges such as high computational costs and difficulties in feature fusion. The ST-ATGCN iteratively employs SA-GC and TSA modules to process the input sequences, outputting separate latent codes for spatial and temporal dimensions to construct the final latent space. This enables the ST-ATGCN to achieve higher computational efficiency and effectively fuse spatio-temporal information during the propagation process, thus reducing the complexity of capturing intricate spatio-temporal relationships. Consequently, it can effectively understand more complex human motions.

Our model was trained and validated on two large-scale motion datasets, NTU-RGB-D 60 and NTU-RGBD-120 [18], while we also constructed datasets NTU-RGB-ER and MCP-ER for the assessment of motion enhancement efficacy. NTU-RGB-ER, artificially created based on the NTU-RGB-D dataset, encompasses motion sequences with errors alongside the ground truth. MCP-ER, on the other hand, was developed using flawed human motion data collected with a real wearable sensor, Sony mocopi [19]. The experimental results indicate that the ST-ATGCN, while ensuring resistance to flawed data in the training sets, can effectively reconstruct and recover motion sequences with errors.

Our contributions can be summarized as follows:

- We designed a novel spatio-temporal attention-based graph neural network, ST-ATGCN, for denoising and enhancing flawed human action sequences. The enhanced understanding of human skeletal structure by the ST-ATGCN allows it to achieve satisfactory results without the need for additional prior knowledge constraints.
- We established direct connections between distant nodes with a learnable shared matrix that addresses the issue of difficult communication between distant nodes with traditional GCNs, significantly improving the efficiency of information transmission between distant nodes.
- We utilized a temporal attention-based module designed to overcome the limitations of a TCN and RNN in processing human motion sequence, such as limited receptive fields and difficulties with long-term memory.
- We conducted training and extensive experiments on two large public human motion datasets, NTU-RGB-D 60 and NTU-RGB-D 120. Additionally, we constructed partially flawed human motion datasets, NTU-RGB-ER and MCP-ER, to validate the motion enhancement effect. The experimental results confirm the effectiveness of our model.

## 2. Related Work

### 2.1. Motion Capture Technologies

Affordable consumer-grade motion capture sensors are broadly classified into three main types: 2D optical sensors [20], depth optical sensors [21], and wearable sensors [22]. Each has unique methodologies and limitations for capturing human motion. Two-dimensional (2D) optical sensors utilize human pose estimation techniques to infer motion [20]. These techniques involve the application of pretrained neural networks that estimate three-dimensional (3D) skeletal joint coordinates from the two-dimensional imagery of the human skeleton [23,24]. Although 2D optical sensors are cheap to access, they encounter limitations due to their reliance on 2D images for estimating 3D models. The quality and quantity of 2D images directly influence the accuracy of 3D human motion data [25,26]. In contrast, depth optical sensors also employ human pose estimation for extracting skeleton information, but they can capture the depth information of the scene [2,27,28]. This capability reduces the errors associated with the direct estimation of 3D models from 2D data. However, this additional functionality comes at a higher cost. Additionally, lower-priced depth sensors exhibit a decrease in accuracy concerning depth data [29]. Wearable sensors are designed to be affixed to key joints of the skeleton. They gather positional data through integrated gyroscopes and synthesize this information to generate human motion data [1,7,9,30]. Although wearable sensors offer direct measurements of movement, they typically cover a limited number of skeleton joints. The number of sensors on low-cost wearable motion capture suits is significantly lower than those in industrial-grade motion capture systems [31]. Consequently, the data for unmonitored joints must often be inferred through algorithms, leading to a mix of direct and estimated data.

### 2.2. Processing Human Motions with GCNs

With the rapid advancement of human pose estimation technology, utilizing extensive skeletal data for the training of human motion models has become feasible. The use of graph convolutional networks (GCNs) for processing human skeletal data has quickly emerged as a research hotspot. The spatio-temporal graph convolutional network (ST-GCN) is the first method to employ spatio-temporal GCNs for human action recognition [11,15], and some researchers have further optimized the ST-GCN model to enhance the recognition accuracy on this basis [12,32]. The ST-GCN employs a single-stream structure to simultaneously process spatio-temporal information, while some studies propose the use of a dual-stream architecture to separately handle spatial and temporal information [13]. Beyond human action recognition, some research has focused on using GCNs for human motion synthesis [14,33]. Degardin et al. proposed a method that combines generative adversarial architectures with GCNs for synthesizing human motions [34]. Converting 2D human skeletal data to 3D has also become a popular topic, facilitating the direct acquisition of 3D human motions from 2D optical motion capture sensors. With the popularity of ChatGPT, studies have also attempted to integrate large language models to provide additional human kinematic prior knowledge for GCN-based methods, thereby enhancing the capability of GCNs in processing human motion data [35].

### 2.3. Human Motion Enhancement Methods

Filtering methods are among the most commonly used techniques for processing sensor data. Employing methods such as Kalman filtering and particle filters to impose additional constraints on human motion data is a common practice [36,37]. Several studies have modified the Kalman filter to achieve better human motion restoration effects [38]. While filtering methods are classic, they also have many limitations, and learning-based approaches have garnered more attention. Methods that improve overall human motion quality by learning human motion manifolds are considered to have good interpretability [39,40]. Then, reconstruction networks based on autoencoders are widely used for denoising tasks, where the processes of data compression and restoration effectively eliminate noise information. Therefore, a considerable number of approaches use autoencoder-based neural network structures for reconstructing low-quality human motion data, thereby obtaining denoised human motion data [40,41,42]. In terms of the temporal dimension, employing models based on RNNs or LSTM to capture the relationships between human motions at different time steps is a common approach [39,41].

## 3. Materials and Methods

**Figure 2:**
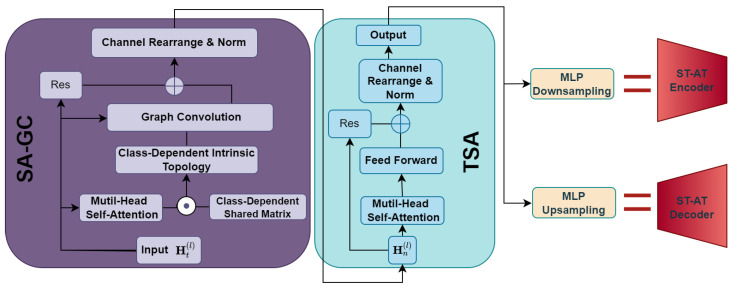
The ST-AT encoder consists of an SA-GC module, a TSA module, and an MLP network for downsampling. The ST-AT decoder is composed of an SA-GC module, a TSA module, and an MLP network for upsampling. RES denotes residual connections, employed to optimize the training of the network.

In this section, we provide a detailed exposition of the construction and design of each submodule within the ST-ATGCN framework. The overarching structures of the most pivotal components, the ST-AT encoder and ST-AT decoder, are illustrated in Figure 2. Furthermore, we delineate the architecture of the model inputs and outputs, alongside the logic of the data transformation. The specific functions of each module, the rationale behind their design, and the issues they aim to address are also discussed. In addition, we elucidate the method of the model’s training and the methodologies employed for evaluation.

### 3.1. Preliminary

The human skeleton can be conceptualized as an undirected graph, denoted as G=(V,E), where V represents the set of key nodes within the human skeleton, denoted as $\left\{v_{1},v_{2},\cdots ,v_{N}\right\}$, and E represents the set of connections between nodes that collectively constitute the structure of the human torso, denoted as $\left\{e_{1},e_{2},\cdots ,e_{K}\right\}$. The features of a motion graph sequence with *T* frames are denoted as $X=\left\{x_{1},\cdots ,x_{T}\right\}\in R^{T\times N\times C}$, where *C* represents the dimensionality of the features.

$H^{\left(l\right)}$ is used to denote the input at each layer in the network, where l represents the index of the network layer. The input for the first layer $H^{\left(0\right)}$, can be expressed as follows:

(1) $$H_{t}^{\left(0\right)}=\mathrm{Linear}\left(x_{t}\right)+PosEmbedding$$

We perform a linear transformation on the original motion data $x_{t}$ at frame t and add a position embedding to obtain the motion embedding.

### 3.2. Unconstrained Human Structure Learning

#### 3.2.1. Limitations of GCNs

The graph convolutional network, as introduced in [32], employs graph convolution techniques to handle data organized in a graph structure. The underlying convolution operation of the GCN can be expressed mathematically as follows:

(2) $$H_{t}^{\left(l+1\right)}=\sigma \left(\hat{A}H_{t}^{\left(l\right)}W^{\left(l\right)}\right).$$

$\hat{A}=D^{-\frac{1}{2}}\left(A+I\right)D^{-\frac{1}{2}}$ is the normalized adjacency matrix, where A is the adjacency matrix, and identity matrix I indicates that each node has a self-connection. D is the diagonal degree matrix of A+I. $W^{\left(l\right)}\in R^{D_{l}\times D_{l-1}}$ denotes the learnable weights in the *l*-th layer. The activation function used is denoted as σ(·). The static adjacency matrix $\hat{A}$ serves as the fundamental source of structural information required for graph convolutions.

**Figure 3:**
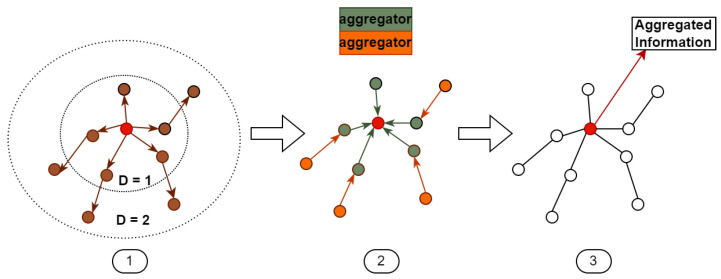
The operation mode of traditional graph convolutional networks (GCNs) primarily encompasses three stages: 1. Sample neighborhood. 2. Aggregate feature information from nearest neighbors. 3. Node information with aggregated features.

The information aggregation in GCNs depends on the input adjacency matrix, which delineates the connections between nodes and their neighbors. In scenarios where the graph structure remains static, the adjacency matrix also remains unchanged. Following the convolution principle of GCNs, each convolution operation enables each node to aggregate information from its neighboring nodes, indicating a positive correlation between the depth of the convolutional layers and the maximum distance reachable by each node from its neighbors, as shown in Figure 3. However, this presents a challenge: as each node aggregates information from all its neighbors while retaining its own information, the information from neighboring nodes becomes diluted with each transmission, and this dilution effect intensifies as the depth of the convolutional layers increases. This implies that in traditional GCNs, the connection between two nodes diminishes as their distance increases, leading to a lesser mutual influence. While this is reasonable for standard graph data, it poses problems when applied to human skeletal structure graphs.

In the context of a human skeleton structural graph, the influence of nodes that are further apart can be greater than that of nearer points. This phenomenon is attributable to the bilateral symmetry inherent in the human skeleton, where, in many motions, information corresponding to symmetric nodes may manifest as either identical or inverse. Examples include the nodes for the left and right elbows, and the left and right ankles. In numerous bodily motions, these nodes share a strong connection, with their inter-node information exchange being more significant than that of closer nodes. Notably, in a human skeleton structure graph, these nodes are distanced farther apart, which diminishes their influence due to a lower information transmission efficiency. Additionally, the excessive distance between these nodes necessitates a greater number of graph convolutional layers to ensure information flow, albeit at a reduced efficiency. This requirement contributes to models becoming more cumbersome and challenging to train, impeding the effective learning of the intrinsic relationships within the human skeletal structure.

To address this issue, we employed the method of constructing an intrinsic topological map of the human skeleton. We established information transmission shortcuts between distant nodes, enabling nodes with strong connections to directly communicate without being constrained by the depth of the convolutional layers.

#### 3.2.2. Self-Attention-Based Graph Convolution

The SA-GC module [43] employs a new self-attention-based parameter matrix. The module comprises two primary components. First is an unconstrained and adaptable shared matrix denoted as $\tilde{A}$. $\tilde{A}$ represents a learnable N∗N matrix capturing shared spatial information across different instances over time. In conventional graph convolutional networks (GCNs), the human structural adjacency matrix $\hat{A}$ serves merely as an initializer for the values within this shared matrix. Notably, the shared matrix $\tilde{A}$ encapsulates the foundational human structural information and subsequently learns the implicit direct relationships between individual skeleton nodes during network propagation. The direct relationship information pertaining to nodes is discerned through the following SA module:

(3) $$\mathrm{SA}\left(H_{t}^{\left(l\right)}\right)=\mathrm{softmax}\left(\frac{H_{t}^{\left(l\right)}W_{K}{\left(H_{t}^{\left(l\right)}W_{Q}\right)}^{T}}{\sqrt{D^{\prime }}}\right)$$

$\mathrm{SA}\left(H_{t}\right)$ is an attention matrix obtained based on the self-attention technique [44]. The matrix $W_{Q}$ serves as the weight matrix employed to linearly transform the input sequence $H_{t}^{\left(l\right)}$ into the query space. Similarly, $W_{K}$ functions as the weight matrix utilized to linearly transform the input sequence $H_{t}^{\left(l\right)}$ into the key space. The parameter $D^{\prime }$ denotes the dimensionality of the key vectors subsequent to the linear transformation. This dimensionality determines the scaling factor during the computation of attention scores.

**Figure 4:**
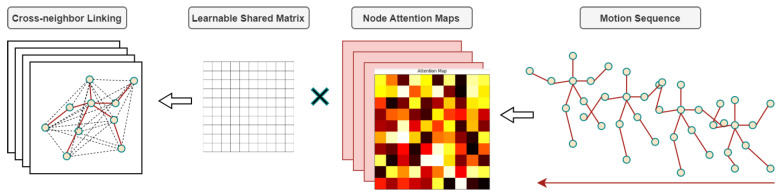
The SA-GC module converts human skeletal nodes in each frame into attention maps. These maps are element-wise multiplied with a learnable shared parameter matrix to capture long-range inter-node relationships across frames.

Attention maps serve as representations to elucidate the correlation or significance between each query element and every other element within a sequence. Where our input comprises skeletal node features at time t, $\mathrm{SA}\left(H_{t}\right)$ essentially delineates the correlation between individual skeletal nodes and all other nodes within the sequence. This explains the direct associative relationships among the nodes previously mentioned. By calculating the dot product between the node attention matrix and shared matrix,

(4) $$\tilde{A}⊙\mathrm{SA}\left(H_{t}\right)\in R^{N\times N},$$

we can obtain a dynamically adaptable adjacency matrix, which is also considered an intrinsic topology of skeleton graphs, as shown in Figure 4. Utilizing an unconstrained learnable matrix instead of the invariant adjacency matrix characteristic of conventional GCN augments the model’s capability to adaptively learn the skeletal structure, while maintaining fidelity to the foundational GCN convolution principles. The structure of an attention map closely resembles that of an adjacency matrix, where each element within the map matrix represents a direct relationship between nodes. By continuously supplying the shared matrix with inter-node relationships from the attention map, the shared matrix can learn to establish shortcuts between two nodes. Specifically, a static adjacency matrix contains values only at positions connecting two nodes by an edge, with all other positions set to zero. In contrast, a learnable shared matrix, through the attention map, can update zeros with newly learned values, representing the weights of direct influence between two nodes. Furthermore, by integrating a multi-head attention mechanism and deploying independent shared matrices tailored for distinct motion categories, We can derive the class-dependent intrinsic topology as follows:

(5) $${\tilde{A}}_{y,m}⊙{\mathrm{SA}}_{m}\left(H_{t}\right)\in R^{N\times N}.$$

A class-dependent intrinsic topology can employ a different shared matrix ${\tilde{A}}_{y}$ for different human motions. y is the class label of the input motion sequence and m is the number of heads in a multi-head self-attention structure. ⊙ is the element-wise product.

The unconstrained shared matrix enables autonomous learning and creates efficient connections between distant nodes. The entire feed-forward propagation in class-dependent SA-GC is recursively conducted as follows:

(6) $$H_{t}^{\left(l+1\right)}=\sigma \left(\sum_{m=1}^{M}\left({\tilde{A}}_{y,m}^{\left(l\right)}⊙SA_{m}\left(H_{t}^{\left(l\right)}\right)\right)H_{t}^{\left(l\right)}W_{m}^{\left(l\right)}\right).$$

The y label can be used to customize the adjacency matrix for different motions. Nonetheless, for a universal motion enhancement model, we can employ the same shared matrix across all motion categories to learn general motion patterns unsupervised. Specifically, for supervised training regimes, it is possible to establish distinct shared matrices ${\tilde{A}}_{y}$ for each action category, creating a set of shared matrices $\left\{{\tilde{A}}_{1},{\tilde{A}}_{2},\cdots ,{\tilde{A}}_{Y}\right\}$. Consequently, different actions are propagated through disparate structural logic. In contrast, in unsupervised training scenarios, a single shared matrix $\tilde{A}$ is employed for all types of actions to encapsulate a more universal logic of motion. Notably, due to the guidance provided by action labels in supervised training, the data requirement for each type of action is comparatively lower. However, to prevent overfitting to a particular action, unsupervised models necessitate a broader variety and greater quantity of training data.

### 3.3. Temporal Self-Attention Layer

The TCN excels in handling dense keyframe motion sequences but falters with sparse inputs [12]. While employing multiple convolutional kernels of varying sizes can mitigate this issue, it still necessitates the manual tuning of multiple hyperparameters. The fixed receptive field size limits model flexibility. While RNNs can accommodate longer sequence lengths by adjusting their memory cycles, the computational speed and memory consumption correspondingly increase with the sequence length [16]. Furthermore, longstanding issues such as gradient vanishing or exploding gradients render the model convergence challenging.

Correspondingly, we adopted a multi-head TSA module instead of a TCN or RNN structure. The multi-head attention mechanism allows the model to process the motion in each frame while aggregating the motion information of all other frames in the sequence. The self-learning attention map enables the ST-ATGCN to adeptly handle sparser and longer sequences, unrestricted by the size of the receptive field or the computational cost. And it is evident that the attention-based generation model yields superior results in comparison to the TCN or RNN. To ensure that the output $H_{t}\in R^{N\times C}$ of the SA-GC module conforms to the logic of the TSA calculation, it is imperative to rearrange the entire motion sequence $H_{T}\in R^{T\times N\times C}$ into $H_{N}\in R^{N\times T\times C}$. Subsequently, this will allow us to derive the input $H_{n}\in R^{T\times C}$ required for TSA. This transformation connects various modules within the ST-ATGCN. The computation of the attention map for the temporal dimension can be expressed as follows:

(7) $$\mathrm{TSA}\left(H_{n}^{\left(l\right)}\right)=\mathrm{softmax}\left(\frac{H_{n}^{\left(l\right)}W_{K}{\left(H_{n}^{\left(l\right)}W_{Q}\right)}^{T}}{\sqrt{D^{\prime }}}\right)W_{V}.$$

The weight matrix $W_{V}$ serves the purpose of linearly transforming the value component of the input sequence, subsequently mapping it to a distinct representation space. It should be noted that in the spatial dimension, we process the features of all nodes in each frame, while in the temporal dimension, the processing involves the extraction of features from the same node across all frames in the motion sequence.

### 3.4. Latent Space

Data compression and restoration are crucial steps in error correction models. The encoder module compresses the input motion sequences, learning to discard erroneous information while retaining essential correct details. Conversely, the decoder module focuses on reconstructing complete and accurate motion sequences from the compressed latent code. During each iteration, the encoder employs the SA-GC module and the TSA module followed by downsampling to compress the data information. In contrast, the decoder executes upsampling to restore the accurate sequence. We implemented two self-learning multi-layer perceptrons, MLP_ds_ and MLP_us_, for upsampling and downsampling motion sequences post-TSA layer processing as follows:

(8) $$H_{t}^{\left(l+1\right)}={\mathrm{MLP}}_{\mathrm{ds}}\left(H_{2t-1}^{\left(l\right)},H_{2t}^{\left(l\right)}\right).$$

(9) $$\left[H_{2t-1}^{\left(l+1\right)},H_{2t}^{\left(l+1\right)}\right]={\mathrm{MLP}}_{\mathrm{us}}\left(H_{t}^{\left(l\right)}\right).$$

where *t* is the frame index, and *l* denotes the current layer of the perception network. For a comprehensive motion sequence denoted as $H_{t}$ with a length T, following the processes of downsampling and upsampling, its length becomes T/2 and 2T, respectively.

Considering that the learnable parameter matrices updated through attention-based mechanisms contain only non-negative values, and that the essence of GCN propagation is a process of information aggregation, we adopted a method that separately takes the maximum values along the temporal and spatial dimensions before concatenating them to construct the latent space. This approach, which contrasts with the mean-based method used in some studies, more effectively aggregates key node and key frame information during the downsampling process. Let the final output of the encoder be denoted as $X^{\left(L\right)}\in R^{C\times T\times N}$, where *T* represents the total number of frames after downsampling. We can obtain a fusion latent code that integrates the spatial and temporal dimensions as follows:

(10) $$Z=\mathrm{Concat}\left(\max_{i=1}^{t}X_{:,i,:}^{\left(L\right)},\max_{j=1}^{n}X_{:,:,j}^{\left(L\right)}\right).$$

The construction of this latent space ensures, to a certain extent, the secondary fusion of temporal and spatial information, while also circumventing the commonly encountered challenges of information fusion in dual-stream models.

### 3.5. Training and Evaluation

#### 3.5.1. Training Objective

The basic training objective of the ST-ATGCN is to reconstruct the input X into XGT. Serving as a model for human motion rectification, mere reconstruction training cannot guarantee the effective correction and enhancement of human motions. It necessitates the formulation of a well-structured training dataset to endow the model with the capability to identify erroneous motions. The construction of the training set input comprises X, which is a combination of $X_{N}$ and $X_{E}$, where $X_{N}$ represents the set of normal motions, and $X_{E}$ encompasses the set of erroneous motions. The target output for the training set XGT is a combination of set $X_{\mathrm{NGT}}$ and set $X_{\mathrm{EGT}}$, where $X_{\mathrm{NGT}}$ equals $X_{N}$, and $X_{\mathrm{EGT}}$ corresponds to the ground truth for $X_{E}$. This configuration ensures that the model can accurately restore normal human motions while identifying and rectifying motions with errors.

Let $\hat{X}$ denote the output of our model. Then, the training objective of our model is shown as follows:

(11) $$L_{rec}=\frac{1}{NT}\sum_{n=1}^{N}\sum_{t=1}^{T}\left({∥{\mathrm{xgt}}_{t}^{n}-{\hat{x}}_{t}^{n}∥}_{1}\right),.$$

where ${\mathrm{xgt}}_{t}^{n}$ represents the feature value of the n-th node at time t for a single sample in XGT, while ${\hat{x}}_{t}^{n}$ represents the feature value of the n-th node at time t for a single sample in $\hat{X}$.

**Figure 5:**
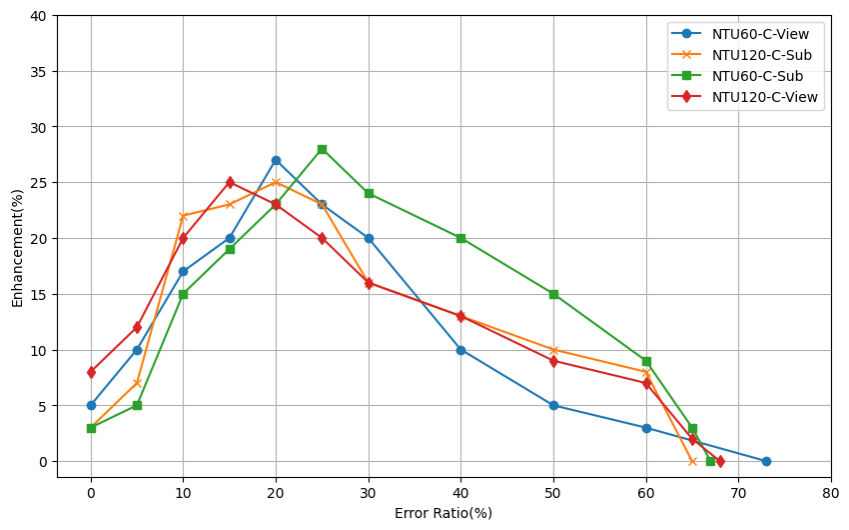
The impact of the proportion of flawed to normal motion data in the training set on improving motion sequence quality with the ST-ATGCN model is shown. The results from training on the NTU-RGB-D 60 and NTU-RGB-D 120 datasets, using cross-subject and cross-view benchmarks, are denoted by NTU60-C-View, NTU120-C-Sub, NTU60-C-Sub, and NTU120-C-View, respectively.

The proportions of $X_{N}$ and $X_{E}$ in the training set have a significant impact on the model’s training performance. A disproportionately high ratio of $X_{N}$ leads to model overfitting, transforming it into a mere reconstruction network, while an excessive ratio of $X_{E}$ results in difficulties in convergence and the erroneous reconstruction of correct motions. We experimented with various combinations of the $X_{N}$ and $X_{E}$ proportions, with the results illustrated in Figure 5.

#### 3.5.2. Evaluation Methodology

Most human motion datasets comprise motion data collected from different individuals at various angles. Prior to a unified evaluation, it is necessary to perform pose matching operations to normalize data across different scales for easier assessment. In this study, we employed Procrustes Superimposition to standardize human motions [45,46]. Due to variations in sensor coordinate systems, the scale of human skeleton data in the world coordinate system also varies. Procrustes Superimposition is a method that allows for scaling and rotating the target without altering its original shape. We employed this method to standardize the output human skeleton data, enabling evaluations under a unified data scale. We also designed an accuracy-based evaluation method, which is fundamentally similar to other methods that measure the absolute distance between nodes. However, it employs a standardized benchmark as the base for correctness, facilitating a more intuitive understanding of the model’s enhancement effects on flawed data.

The validation of the ST-ATGCN model encompassed two parts. Firstly, the verification of the model’s reconstruction capability, which serves to ascertain the model’s proficiency in reconstructing accurate motions. This part was used to assess the accuracy of the joint coordinates. The fundamental ability of the ST-ATGCN model is the precise recognition and reconstruction of error-free motions. Let $C=\left\{c_{1},\cdots ,c_{K}\right\}$ represent the ground truth features of each human skeleton node and $\hat{C}=\left\{{\hat{c}}_{1},\cdots ,{\hat{c}}_{K}\right\}$ represent the features output by the ST-ATGCN. Then, the accuracy of reconstructing features can be defined as

(12) $${\mathrm{Acc}}_{f}=\frac{1}{K}\sum_{k=1}^{K}\left(1-\frac{\left|c_{k}-{\hat{c}}_{k}\right|}{c_{k}^{D}}\right).$$

Given that we standardized the features across each dimension through pose matching, we utilize $c_{k}^{D}$ as the basis for the accuracy of each feature dimension, where $c_{k}^{D}$ represents the difference between the maximum and minimum values of the k-th dimensional feature across all nodes, in the current ground truth motion sequence.

In general human skeleton models, a feature typically includes the coordinates of the joints. Therefore, besides the accuracy of the feature, we can also assess the overall similarity between the output and the real motion by calculating the joint angles. Unlike absolute coordinates, the similarity of joint angles can further verify whether the model has understood the intrinsic logic of the human skeleton structure across different dimensions. Let $\hat{J}$ represent the output joint angles and $\hat{J}$ represent the ground truth of joint angles. The similarity of joints can be defined as

(13) $${\mathrm{Acc}}_{j}=\frac{1}{S}\sum_{s=1}^{S}\left(1-\frac{\left|j_{s}-{\hat{j}}_{s}\right|}{2\pi }\right)$$

where *S* denotes the total number of joints within the human skeletal structure. Given that the calculation of joint angles employs the radian system, the basis for the accuracy assessment is 2π.

The second part of the validation involves assessing the model’s capability to enhance and correct motion data that contain errors. In this phase, the model takes erroneous motion sequence as input, and the features of the erroneous node in the erroneous motion sequence are denoted as $\mathrm{Cer}=\left\{{\mathrm{cer}}_{1},\cdots ,{\mathrm{cer}}_{K}\right\}$. The accuracy of the erroneous motion data relative to ground truth motion data for each erroneous node can be represented as follows:

(14) $${\mathrm{Err}}_{f}=\frac{1}{K}\sum_{k=1}^{K}\left(1-\frac{\left|c_{k}-{\mathrm{cer}}_{k}\right|}{c_{k}^{D}}\right).$$

The accuracy of the enhanced motion data relative to the ground truth motion data for each erroneous node is defined as

(15) $${\mathrm{Enc}}_{f}=\frac{1}{K}\sum_{k=1}^{K}\left(1-\frac{\left|c_{K}-{\hat{c}}_{k}\right|}{c_{k}^{D}}\right).$$

The calculation of ${\mathrm{Enc}}_{f}$ is identical to that of ${\mathrm{Acc}}_{f}$, with the distinction that the latter involves computation over the entire set of normal nodes, whereas the former restricts computation solely to the set of erroneous nodes. Hence, the enhancement effect on each erroneous node can be represented as ${\mathrm{Enc}}_{ef}={\mathrm{Enc}}_{f}-{\mathrm{Err}}_{f}$.

These two assessment sections were, respectively, utilized to validate the model’s capability in reconstructing correct motions as well as enhancing flawed motions.

## 4. Experiment

Through multiple distinct experiments, we validated the performance of the ST-ATGCN, including its capability to accurately reconstruct normal motion sequences and repair flawed motion sequences. Our model was developed and trained utilizing the PyTorch framework. The experimental workstation was equipped with dual NVIDIA RTX 3090 GPUs, an Intel Core i9-9920X CPU, and 64 GB of DDR4 memory. Depending on the specific requirements of each experiment, we set different hyperparameters for model training. Among these, the batch size of 20 yielded the best average training results. After 800 training epochs, a notable decline in convergence speed was observed, indicating that the model had achieved optimal training and testing outcomes at this stage. In the experiment, we employed a five-layer SA-GC&TSA network, which was applied to both the encoder and decoder segments. Throughout the training process, a fixed learning rate of 0.005 was utilized.

### 4.1. Dataset

In this study, we utilized four datasets. The NTU-RGB-D 60 and NTU-RGB-D 120, maintained by Nanyang Technological University, are human motion datasets containing 60 and 120 distinct human motions, respectively, with a total of 57,600 and 114,480 samples. For each motion sample, the NTU-RGB-D datasets provide image data collected using 2D optical sensors, depth image data captured using depth optical sensors, 3D human skeleton data obtained through human pose estimation techniques, and data from infrared sensors. Notably, the 3D skeletal data are structured using a framework of 25 skeletal nodes. The node feature includes the XYZ coordinates of the node in a three-dimensional space. These datasets primarily serve for model training, offering fundamental insights into the reconstruction and repair of human skeletal sequences through the ST-ATGCN learning approach, thereby aiding the model in comprehending the logic behind human motions.

The NTU-RGB-ER is a proprietary dataset of human motion defects, derived through selection and manual modification from the NTU-RGB-D dataset. It is noteworthy that the 3D skeleton data within the NTU-RGB-D dataset inherently contain a minimal amount of erroneous data, an issue stemming from the unavoidable inaccuracies of the sensors involved. In utilizing the additional types of corresponding data they provide, it is feasible to obtain the ground truth for these erroneous motions, facilitating the construction of the dataset. However, due to the scarcity of such erroneous data, artificial defect data were also generated by randomly introducing noise into the correct motion data through manual manipulation. It should be noted that our NTU-RGB-ER dataset is comprised of three sub-datasets. These sub-datasets maintain uniformity in terms of the types of motions, the number of samples, the number of noised nodes, and their distribution. The only variation is in the magnitude of the error, which was set to three different levels.

The MCP-ER dataset is an error-prone motion data collection acquired through the wearable motion capture sensor SONY mocopi. The mocopi system comprises six sensors, designated to be worn on the two wrists, two ankles, head, and waist, respectively. Each node’s data encompass its three-dimensional spatial coordinates as well as the quaternion representation of the node’s rotational coordinate system. Although only six points of motion data are directly captured by these sensors, the mocopi’s internal algorithms generate additional skeletal data points, resulting in a dataset that surpasses the direct sensor captured. Over time, significant inaccuracies have been observed in the motion data output by the mocopi. We collected 300 erroneous samples across 15 motion types to construct the MCP-ER dataset. The primary objective of both the NTU-RGB-ER and MCP-ER datasets is to validate the effectiveness of the ST-ATGCN in correcting and enhancing erroneous motion sequences.

### 4.2. Motion Reconstruction Evaluation

Reconstructing normal motion sequences is a fundamental capability of autoencoder-based models, serving as a prerequisite for error motion correction. In this section of the experiment, we evaluated the reconstruction abilities of the ST-ATGCN on the NTU-RGB-D 60 and NTU-RGB-D 120 datasets, respectively. Our experiments adhered to two benchmarks recommended by the NTU-RGB-D dataset: (1) the cross-subject benchmark, which involves using motion data collected from a sample of subjects for training and the remaining subjects’ data for model evaluation and testing; and (2) the cross-view benchmark, which employs motion data captured from certain optical sensor angles as the training set, with the data from the remaining angles used for testing and evaluating the model.

In our comparative analysis, we selected methods that adhere to two criteria: (1) employing GCNs for processing human motion data and (2) capable of handling temporal sequences of motions. While GCNs represent the mainstream approach for processing human skeletal motion data, methods focused solely on reconstructing motion sequences based on GCNs are rare. We identified leading methodologies and constructed networks with an autoencoder structure based on their modules for comparison. Some methods included only an encoder design; we obtained the decoder through the reversed encoder structure. Experimental results on the NTU-RGB-D 60 dataset are presented in Table 1.

The spatio-temporal graph convolutional network (ST-GCN) [11] represents the inaugural approach utilizing a spatio-temporal-based GCN for the analysis of human motion sequences, serving as a baseline for comparison in our study. InfoGCN employs the SA-GC module combined with a TCN for processing human motion sequences [12], while the 2s-AGCN introduces a dual-stream architecture [13]. The SA-GCN method, on the other hand, leverages RNNs for sequence processing [14]. Yhe AA-GCN is an autoencoder-aided GCN framework [47]. As demonstrated in Table 1, the ST-ATGCN exhibits a commendable performance in the task of motion sequence reconstruction. On C-subject benchmark, it achieved a joint coordinate accuracy of 98.09% and a joint angle accuracy of 98.23%. Moreover, on the C-View benchmark, it recorded a joint coordinate accuracy of 97.38% and a joint angle accuracy of 97.55%. It is observed that the experimental outcomes under multi-angle conditions are somewhat inferior to those involving multiple subjects, attributable to the additional complexity of handling data across varying world coordinate systems, which undeniably imposes extra burdens on the model beyond recognizing human structural anatomy. Experimental findings further reveal that the ST-ATGCN significantly outperforms other methodologies in the joint angle reconstruction accuracy, underscoring its superior capability in comprehending the multidimensional structure of the human skeleton beyond merely focusing on the absolute numerical values of node coordinates.

Table 2 presents the reconstruction experiment results on the NTU-RGB-D 120 dataset, which, compared to those on the NTU-RGB-D 60 dataset, contains a larger variety of motion types and a higher volume of samples. This scenario poses a greater challenge to the model’s learning and memory capabilities. The experimental outcomes indicate decreases in the reconstruction performances of all methods to varying degrees under these conditions. However, models employing RNNs exhibit a smaller decline in performance compared to those based on TCNs. Notably, our model, which incorporates a temporal attention layer, namely, the ST-ATGCN, demonstrates the minimal performance degradation when dealing with a more diverse set of samples and motion types, without any alteration in model size. This resilience can be attributed to the learning capabilities of attention-based models and the parallel computation structure, which enables handling larger datasets effectively. Although the joint coordinate accuracy of the SA-GCN slightly surpasses that of the ST-ATGCN on the C-subject benchmark, the ST-ATGCN maintains a lead in joint angle accuracy. This advantage stems from the ST-ATGCN’s more generalized understanding of human skeleton structure.

### 4.3. Motion Enhancement Evaluation

In this section, we primarily validate the enhancement and correction effects of the ST-ATGCN model on flawed motion sequences. We selected three different architectural enhancement methods as our baseline for comparison. These include TPE-DE, which employs a Tobit particle filter for motion enhancement [38]; BRA-P, an autoencoder model based on LSTM [41]; and a STRNN, an RNN model that models human motion by learning human flow dynamics [39]. These methods represent the mainstream frameworks for processing human motion data. Additionally, we utilize three distinct flawed motion datasets to assess the enhancement effects of each method on motion, namely, NTU-RGB-ER-A, NTU-RGB-ER-B, and NTU-RGB-ER-C. These subsets of the self-compiled flawed motion dataset NTU-RGB-ER exhibit identical types of motions, equal sample sizes, identical numbers of noised nodes, and uniform distributions of noised nodes. The distinction lies in the magnitude of the error associated with the noised nodes, with their noised data’s similarity to the ground truth motion data being approximately 85%, 75%, and 65%, respectively. This similarity measure, as defined by (14) in the previous text, represents the accuracy of erroneous data relative to the ground truth data for each noised node. The results of the experiment are presented in Table 3.

**Figure 6:**
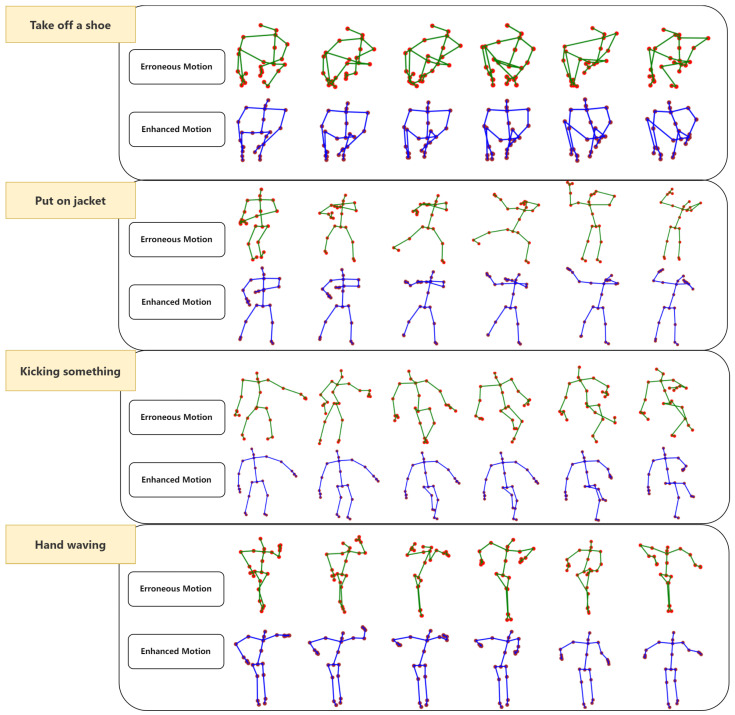
Visualization results of motion enhancement on selected motions from NTU-RGB-ER dataset using ST-ATGCN.

The results from the table demonstrate that our ST-ATGCN model achieved significant improvements across three datasets of flawed motion at varying degrees, closely rivaling the ST-RNN model. However, it is noteworthy that the ST-RNN, by adopting a method of learning human motion manifolds, essentially provides the training model with additional a priori knowledge. Moreover, as the proportion of flawed motion data increases, there is a noticeable enhancement in the average ${\mathrm{Enc}}_{f}$ for all methods. Yet, the variation in ${\mathrm{Enc}}_{f}$ between filtering approaches and neural network-based methods differs with the increase in flawed data. Specifically, filtering methods exhibit a pronounced decline in ${\mathrm{Enc}}_{f}$ with a reduced ${\mathrm{Err}}_{f}$, marked at 91.45%, 85.88%, and 78.58%, indicating that their performance is significantly impacted by the increased quantity and error margin of the flaws. This is attributed to the exacerbation of data fluctuation, rendering the filtering more challenging due to its reliance on all noise data. Conversely, neural network-based methods show more stability in ${\mathrm{Enc}}_{f}$ changes; for instance, the STRNN’s ${\mathrm{Enc}}_{f}$ readings of 92.45%, 91.87%, and 89.43% closely approach their maximum ${\mathrm{Enc}}_{f}$. This stability is believed to be due to autoencoder-based models possessing superior capabilities in extracting key information and eliminating noise, coupled with an inherent understanding of the human skeleton structure, making neural network-based methods more resilient. We selected several sequences of motions with imperfections and visualized them after enhancement through various methods, as illustrated in Figure 6.

Overall, the ST-ATGCN exhibited ${\mathrm{Enc}}_{f}$ scores of 95.66%, 93.32%, and 90.64% on the NTU-RGB-ER-A, -B, and -C datasets, respectively, demonstrating enhancement effects of 8.95%, 17.95%, and 26.30% on flawed motion data. Generally, learning-based methods showed a higher performance baseline in restoring normal human motion data. However, in the context of NTU-RGB-ER-A, the good ${\mathrm{Enc}}_{f}$ performance of TPE-DE, 91.45%, suggests that filtering methods also yield commendable results under scenarios of minimal flawed data and mild noise fluctuation. Given the significantly higher computational efficiency of filtering methods compared to learning-based approaches, filtering methods may offer greater cost-effectiveness in specific application contexts.

In Table 4, we show the enhancement effects on specific motion type datasets, where our flawed motion data were selected from the NTU-RGB-ER-B dataset. The dataset exhibited an ${\mathrm{Err}}_{f}$ value around 75%, aligning with realistic scenarios requiring motion data enhancement. We randomly selected eight different motion types and calculated the ${\mathrm{Enc}}_{f}$ and ${\mathrm{Enc}}_{ef}$ for various methods across these motions.

**Figure 7:**
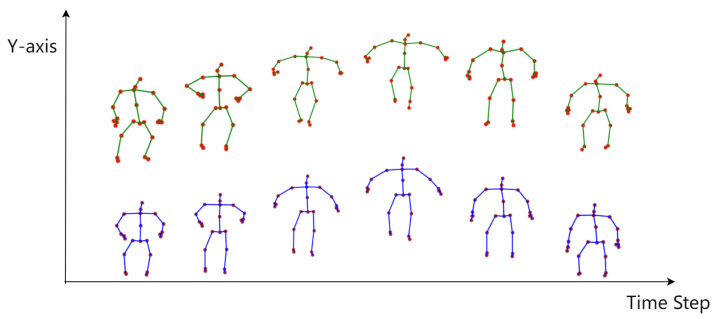
The motion of jumping varies over time on the Y-axis. The upper sequence represents a slightly erroneous jumping motion sequence, while the lower sequence is the output of the ST-ATGCN.

The ST-ATGCN achieved the best enhancement results in most motions, with only slight inferiority to the STRNN in the motions “Throw” and “Thumb Up,” by a margin of approximately 1% in ${\mathrm{Enc}}_{f}$. Notably, the motions “Stand Up,” “Sit Down,” and “Jump Up” require attention. The visualization of the reconstructed motion sequence after denoising for “Jump Up” is shown in Figure 7.

In these motions, both the TPE-DE and ST-ATGCN performed well, whereas the BRA-P and STRNN showed relatively weakened enhancement effects. Specifically, in the “Jump Up” motion, the ${\mathrm{Enc}}_{f}$ values for the BRA-P and STRNN were only 79.63% and 85.32%, respectively, significantly lower than their average enhancement effects on other motions. We found that the BRA-P and STRNN are insensitive to the movement of the human body along the Y-axis, ignoring the displacement on this axis while maintaining a reasonable overall motion shape. In contrast, the ST-ATGCN and TPE-DE did not exhibit this issue.

The sensitivity of the ST-ATGCN along the Y-axis benefits from its SA-GC module’s understanding of the human body structure and the TSA module’s sensitivity to temporal sequence variations. Compared to RNN-based models, which generally consider only preceding time step information, the temporal attention-based TSA module possesses a global perspective of the entire motion sequence. This difference leads to RNN-based models potentially correcting minor overall displacement inaccuracies during the step-by-step correction process. Furthermore, the lack of training data featuring overall displacement along the Y-axis also contributes to this outcome. Unlike learning-based methods, which may be influenced by the training dataset, the filter-based TPE-DE does not arbitrarily correct overall displacement data.

**Figure 8:**
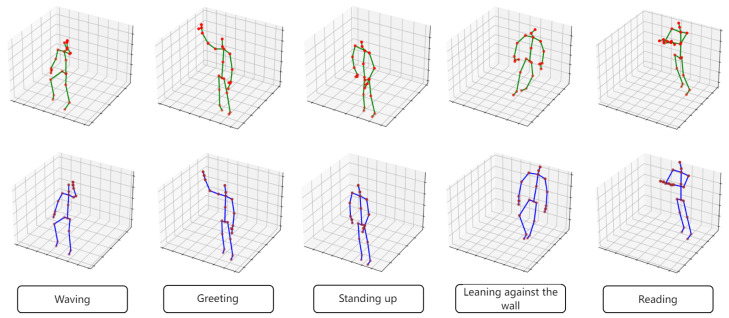
Selected visualization results of motion enhancement on our MCP-ER dataset using ST-ATGCN. The above and below lines respectively show the results before and after enhancement.

After validating on the NTU-RGB-D-ER dataset, we further conducted motion sequence denoising experiments on the MCP-ER dataset, which was collected using a low-cost motion capture sensor, Sony Mocap. In this set of experiments, the primary discrepancies in the flawed motion data that we addressed were twofold: (1) the error between the automatically supplemented human joint coordinates by Mocap and the ground truth and (2) the noise accumulated due to prolonged motion capture by the sensor. Visual results of the experiments are presented in Figure 8.

The noise in the MCP-ER dataset primarily manifests as jitter in movements and some unnatural postures. The experimental results demonstrate that, following reconstruction through the ST-ATGCN, the fluctuating motion sequences become smoother, and ergonomically implausible postures are rendered more natural. This enhances the overall visual appeal of the motion capture results, ensuring that the audience does not experience discomfort upon visualization.

## 5. Conclusions and Future Work

In this paper, we introduced the ST-ATGCN, an autoencoder-based model designed to correct and enhance human motion data that contain errors or imperfections. Our primary focus was on understanding the logic of human motion and the structure of the human skeleton without relying on additional prior knowledge as constraints, thereby improving our ability to process human motion data. We improved upon traditional GCN-based methods from two directions. First, in the spatial dimension, we established direct connections between nodes to overcome the inefficiency of information transmission in GCNs. Second, in the temporal dimension, we adopted an attention-based method for processing time series, replacing TCN-based methods that struggle with sparse time series and RNN-based methods that have difficulty memorizing long-term information and considering complete sequences. Our experimental results demonstrate that the ST-ATGCN can effectively understand the construction of human motions. It can accurately correct abnormalities, such as unnatural angles in individual joints, including reverse joints. The model’s ability to recognize joint angle anomalies with minimal error rates indicates a higher-dimensional understanding of the human skeletal structure, rather than merely learning variations in node coordinate values. For continuous time parts with substantial errors, where it is challenging to obtain effective cues from nearby key frames, the ST-ATGCN outperforms RNN-based or TCN-based methods, a benefit we attribute to TSA’s global perspective.

Although theh ST-ATGCN’s capability in enhancing human motion data was validated, we identify several issues that require resolution, with the integration of temporal and spatial information being the most critical. While we attempted to separate and reintegrate spatio-temporal information through rearrangements and maximum sampling operations, we believe that the construction of the latent space can be further optimized. Although single-stream spatio-temporal models typically fuse information across different dimensions during the encoder and decoder data propagation process, a well-defined latent space can still enhance the overall model performance. In future work, we will explore decoupling and recombining the spatial and temporal latent codes to further eliminate redundancy between them, thereby achieving a more optimized latent space. Another issue concerns the real-time scenarios of motion enhancement methods. Although the speed of learning-based methods has gradually increased, they still do not match the processing speed of filtering methods, making them unsuitable for scenarios with high real-time requirements, such as virtual live broadcasting and real-time motion correction. Additionally, improvements in speed are significantly influenced by the cost of hardware equipment. In future research, we will also focus on enhancing the simplicity of the modules and reducing redundancy to increase the overall processing speed of the models.

## Figures and Tables

**Table 1:** The results of the ST-ATGCN and other GCN-based methods in reconstructing normal motion data on the NTU-RGB-D 60 dataset.

Method	Avg Accuracy (%)			
	C-Subject		C-View	
	${\mathrm{Acc}}_{f}$	${\mathrm{Acc}}_{j}$	${\mathrm{Acc}}_{f}$	${\mathrm{Acc}}_{j}$
ST-GCN	90.78%	91.47%	89.58%	88.98%
SkeletonVAE	93.31%	93.89%	91.03%	90.72%
InfoGCN	93.58%	94.11%	91.28%	93.49%
2s-AGCN	96.82%	96.73%	95.31%	95.62%
AA-GCN	96.66%	97.08%	95.53%	94.33%
SA-GCN	97.78%	97.14%	97.29%	96.17%
ST-ATGCN (Ours)	**98.09%**	**98.23%**	**97.38%**	**97.55%**

Note: The best results in each metric are bolded.

**Table 2:** The results of the ST-ATGCN and other GCN-based methods in reconstructing normal motion data on the NTU-RGB-D 120 dataset. The bolded data represent the best results.

Method	Avg Accuracy (%)			
	C-Subject		C-View	
	${\mathrm{Acc}}_{f}$	${\mathrm{Acc}}_{j}$	${\mathrm{Acc}}_{f}$	${\mathrm{Acc}}_{j}$
ST-GCN	87.78%	89.22%	85.32%	84.28%
SkeletonVAE	90.79%	90.13%	89.05%	91.17%
InfoGCN	92.48%	93.95%	91.99%	93.10%
2s-AGCN	94.61%	94.78%	96.31%	95.24%
AA-GCN	96.07%	97.82%	94.13%	94.64%
SA-GCN	**97.32%**	96.28%	95.77%	94.25%
ST-ATGCN (Ours)	97.21%	**98.11%**	**97.03%**	**97.29%**

Note: The best results in each metric are bolded.

**Table 3:** The results of motion data enhancement on our erroneous motion dataset NTU-RGB-ER using the ST-ATGCN and other motion enhancement methods.

DATASET	METHODS								
	TPE-DE			BRA-P		STRNN		ST-ATGCN	
	${\mathrm{Err}}_{f}$	${\mathrm{Enc}}_{f}$	${\mathrm{Enc}}_{\mathrm{ef}}$	${\mathrm{Enc}}_{f}$	${\mathrm{Enc}}_{\mathrm{ef}}$	${\mathrm{Enc}}_{f}$	${\mathrm{Enc}}_{\mathrm{ef}}$	${\mathrm{Enc}}_{f}$	${\mathrm{Enc}}_{\mathrm{ef}}$
NTU-RGB-ER-A	86.71%	91.45%	4.74%	89.12%	2.41%	92.45%	5.74%	**95.66%**	**8.95%**
NTU-RBG-ER-B	75.37%	85.88%	10.51%	88.21%	12.84%	91.87%	16.50%	**93.32%**	**17.95%**
NTU-RGB-ER-C	64.34%	78.58%	14.24%	86.30%	21.96%	89.43%	25.09%	**90.64%**	**26.30%**

Note: The best results in each metric are bolded.

**Table 4:** The results of enhancing specific motions in the erroneous motion dataset NTU-RGB-ER using the ST-ATGCN and other motion enhancement methods.

Motion	METHODS								
	TPE-DE			BRA-P		STRNN		ST-ATGCN	
	${\mathrm{Err}}_{f}$	${\mathrm{Enc}}_{f}$	${\mathrm{Enc}}_{\mathrm{ef}}$	${\mathrm{Enc}}_{f}$	${\mathrm{Enc}}_{\mathrm{ef}}$	${\mathrm{Enc}}_{f}$	${\mathrm{Enc}}_{\mathrm{ef}}$	${\mathrm{Enc}}_{f}$	${\mathrm{Enc}}_{\mathrm{ef}}$
Stand Up	75.36%	88.42%	13.06%	88.01%	12.65%	91.73%	16.37%	**93.66%**	**18.30%**
Sit Down	74.19%	88.71%	14.52%	89.73%	15.54%	89.45%	15.26%	**91.89%**	**17.70%**
Take Off Jacket	77.64%	85.34%	7.7%	86.81%	9.71%	86.91%	9.27%	**89.68%**	**12.04%**
Throw	73.59%	86.77%	13.18%	90.91%	17.32%	**92.64%**	**19.05%**	91.01%	17.42%
Jump Up	75.68%	89.12%	13.44%	79.63%	3.95%	85.32%	9.64%	**90.43%**	**14.75%**
Thumb Up	71.18%	84.36%	13.18%	86.72%	15.54%	**92.58%**	**21.40%**	92.12%	20.94%
Shake Fist	76.77%	82.45%	5.68%	86.38%	9.61%	90.24%	13.47%	**92.36%**	**15.89%**
Side Kick	75.40%	87.89%	12.49%	91.44%	16.04%	92.50%	17.10%	**94.77%**	**19.37%**

Note: The best results in each metric are bolded.

## Data Availability

A part of the data from the public dataset is available after a reasonable request is made to author T.H. The other private data are not available due to privacy reasons.
